# Supplementary material for: Radiation Promotes Acute and Chronic Damage to Adipose Tissue
Source: Int J Mol Sci. 2025 Jun 12;26(12):5626. doi: 10.3390/ijms26125626 (PMC12193394; doi:10.3390/ijms26125626)
Supplement: Supplementary file 1 [file ijms-26-05626-s001.zip › ijms-3656488-supplementary.pdf]

International Journal of
Molecular Sciences

MDPI

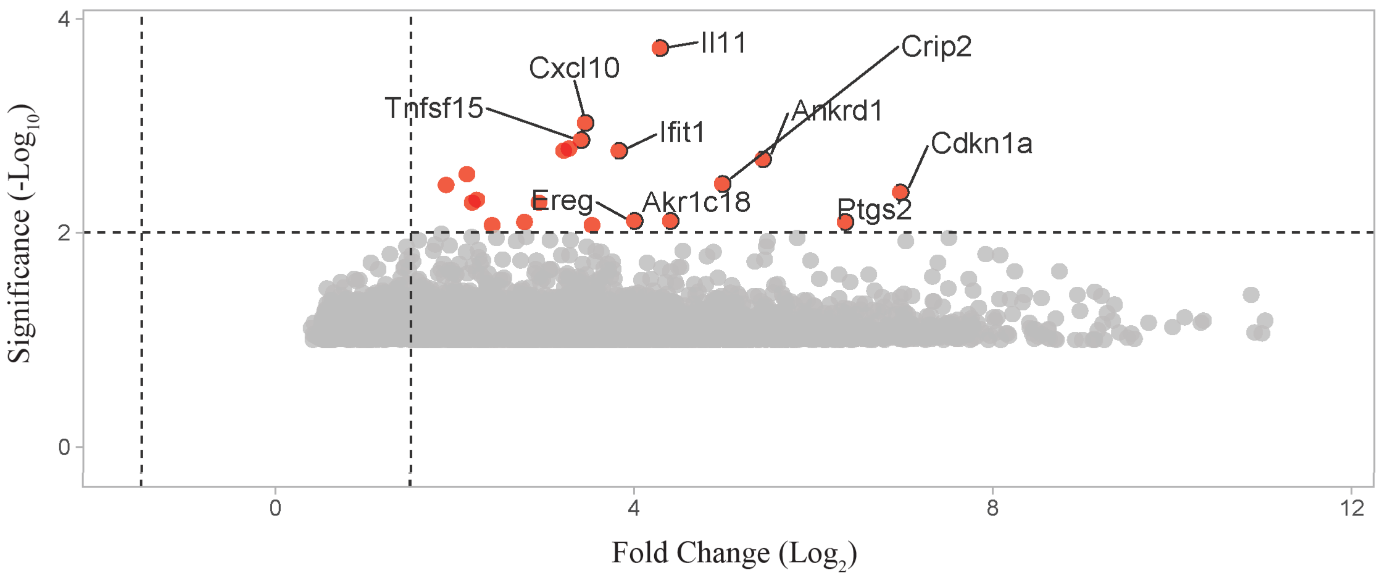

Supplemental Figure S1: Inflammation and senescence-associated genes have elevated expression in irradiated adipocytes. RNA sequencing data was collected from the gonadal fat pads of mice 2 months post-radiation. Red dots represent transcripts that are upregulated in irradiated adipose tissue as compared to unirradiated adipose tissue.

https://doi.org/10.3390/ijms26125626
